# Supplementary figures and images for: Modelling of filamentous phage-induced antibiotic tolerance of P. aeruginosa
Source: PLoS One. 2022 Apr 11;17(4):e0261482. doi: 10.1371/journal.pone.0261482 (PMC9000967; doi:10.1371/journal.pone.0261482)

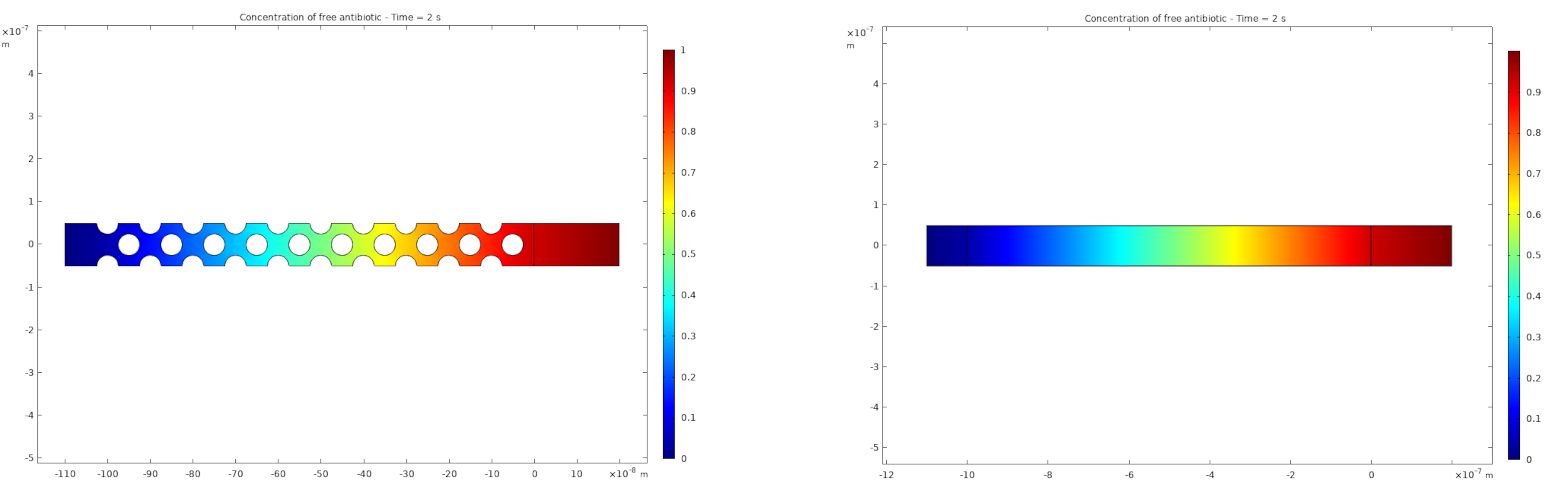

Supplement: S1 Fig — The microscopic model is shown on the left; the homogenised model is shown on the right. The colour scale indicates free antibiotic concentration, circles represent phages. (TIF) [file pone.0261482.s001.tif]

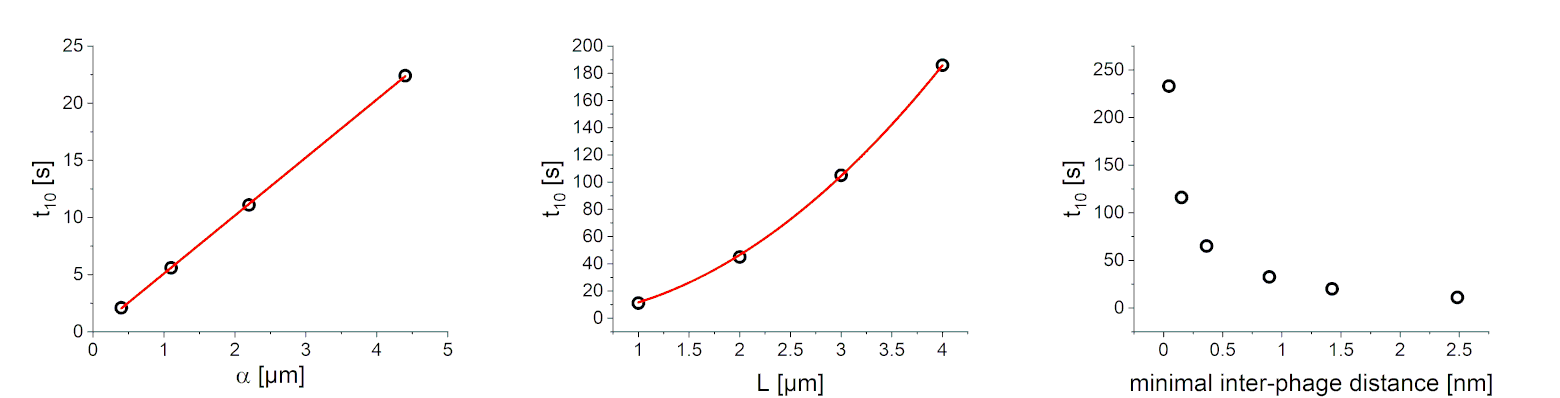

Supplement: S2 Fig — The dependence is shown for the binding equilibrium constant α, with a linear fit indicated by the red line, the tactoid width L, with the red line showing a quadratic fit, and the phage packing density, quantified as the minimal inter-phage distance. (TIF) [file pone.0261482.s002.tif]
