# Supplementary material for: Modelling of filamentous phage-induced antibiotic tolerance of P. aeruginosa
Source: PLoS One. 2022 Apr 11;17(4):e0261482. doi: 10.1371/journal.pone.0261482 (PMC9000967; doi:10.1371/journal.pone.0261482)
Supplement: S1 Appendix — A brief summary of the concept of homogenisation, followed by a description of the homogenised, analytically solvable model. (PDF) [file pone.0261482.s003.pdf]

Appendix S1: Homogenisation

An analytically solvable effective model was developed using homogenisation. This is a mathematical technique used to describe systems in which scale separation occurs, i.e. where the physics at a macroscopic scale and a much smaller microscopic scale can be disentangled. Due to the microscopic structure, the system generally cannot be solved analytically, and numerical modelling is computationally expensive. However, in certain cases one can describe such a system system with effective equations on the macroscopic scale. If the microscopic details of the system are regular, their effect is averaged out and what is left is a much simpler macroscopic structure, governed by effective equations. These can often be solved analytically and give the same results as the microscopic model, in the limit that the microscopic scale becomes infinitesimal.
Homogenisation was applied to the microscopic model, following the work on diffusion in porous media by Allaire et al. (2010) [1]. The liquid crystalline alignment of phages leads to a regular structure which can be considered a lattice of identical unit cells: the microscopic structure to be averaged out. This process is illustrated in Fig S1. Homogenisation leads to the following effective equation:

$$\frac{\partial u(\boldsymbol{x},t)}{\partial t} = \boldsymbol{\nabla} \cdot \left(D^{(\mathrm{eff})}\boldsymbol{\nabla}\right) u(\boldsymbol{x},t), \tag{1}$$

where $D^{(\mathrm{eff})}$ is the effective diffusion coefficient, given by:

$$D^{(\mathrm{eff})} \equiv \frac{\hat{D}}{1+\alpha\frac{|\Gamma|}{|\mathcal{C}|}}. \tag{2}$$

Here $\hat{D}$ is a rescaled diffusion coefficient that takes into account of the physical barrier on diffusion caused by phages. The denominator, instead, represents the effect of adsorption. $|\mathcal{C}|$ and $|\Gamma|$ are the free volume between phages and phage surface in a unit cell, respectively.

This expression can be simplified in the limit of strong adsorption, i.e. $(\alpha|\Gamma|/|\mathcal{C}|) \gg 1$, to

$$D^{(\mathrm{eff})} = \frac{\hat{D}}{\alpha\left(\frac{1}{\alpha}+\frac{|\Gamma|}{|\mathcal{C}|}\right)} \approx \frac{\hat{D}}{\alpha\frac{|\Gamma|}{|\mathcal{C}|}}, \tag{3}$$

thus highlighting that the effective diffusion coefficient is inversely proportional to the equilibrium adsorption coefficient.

References

[1] Allaire G, Brizzi R, Mikelić A, Piatnitski A. Two-scale expansion with drift approach to the Taylor dispersion for reactive transport through porous media. Chem Eng Sci. 2010;65(7):2292–2300. doi:10.1016/j.ces.2009.09.010.
