# Supplementary material for: Modelling of filamentous phage-induced antibiotic tolerance of P. aeruginosa
Source: PLoS One. 2022 Apr 11;17(4):e0261482. doi: 10.1371/journal.pone.0261482 (PMC9000967; doi:10.1371/journal.pone.0261482)
Supplement: S2 Appendix — An account of the derivation of the parameter values from experimental data by Secor et al. (2015) [1] and Tarafder et al. (2020) [2]. (PDF) [file pone.0261482.s004.pdf]

Appendix S2: Parameter estimates

Model parameter values were set as follows. The tactoid width L was estimated from images by Secor et al. (2015) [1] and taken to be $L = 1$ µm. The phage radius a is 3 to 3.5 nm [1]; we use a conservative estimate of 3 nm. We set the unit cell size to be 4 times the phage radius; this value is estimated from Cryo-ET images by Tarafder et al. (2020), showing the configuration of phages inside a tactoid [2].
We take the diffusing antibiotics to be tobramycin, for which the greatest amount of data is available; its diffusion coefficient in an aqueous medium is $D = 15\,\mu\text{m}^2/\text{s}$.
The experiments by Secor et al. (2015) indicate an adsorption of about 30% of the antibiotics by phages in isotropic phase [1], which means $\alpha = 0.4\,\mu\text{m}$. When the phages are nematic, about 70% of antibiotics are adsorbed, which gives $\alpha = 2.2\,\mu\text{m}$.
Since we set $a = 3$ nm and $D = 15\,\mu\text{m}^2/\text{s}$ we have a microscopic diffusion time

$$\tau_D = \frac{a^2}{D} = 6 \times 10^{-7}\,\text{s}, \tag{1}$$

which, assuming that τ_D and τ_κ are of the same order, means $\kappa \simeq 1/\tau_D = 1.7 \times 10^6\,\text{s}^{-1}$. Since κ is the adsorption rate, its value has no influence on the diffusion time, as long as $\kappa \gg 1\text{s}^{-1}$. This is confirmed by the fact that κ does not appear in the homogenised equation. Hence, any large value for κ should give identical results.
A total antibiotic concentration of 200 µg/ml is used in the experiments of Secor et al. (2015) [1] which are comparable to the model presented in this paper. However, since only the relative antibiotic concentration is measured with respect to the average, u and v can be kept dimensionless. Furthermore, we assume that antibiotic adsorption has a negligible effect on the antibiotic concentration in the extracellular matrix surrounding the tactoid. Hence, we take the free antibiotic concentration at the outer tactoid edge to be $u = 1$.

References

[1] Secor PR, Sweere JM, Michaels LA, Malkovskiy AV, Lazzareschi D, Katznelson E, et al. Filamentous bacteriophage promote biofilm assembly and function. Cell Host Microbe. 2015;18(5):549–559. doi:10.1016/j.chom.2015.10.013.

[2] Tarafder AK, von Kügelgen A, Mellul AJ, Schulze U, Aarts DGAL, Bharat TAM. Phage liquid crystalline droplets form occlusive sheaths that encapsulate and protect infectious rod-shaped bacteria. PNAS. 2020;117(9):4724–4731. doi:10.1073/pnas.1917726117.
