# Supplementary material for: Modelling of filamentous phage-induced antibiotic tolerance of P. aeruginosa
Source: PLoS One. 2022 Apr 11;17(4):e0261482. doi: 10.1371/journal.pone.0261482 (PMC9000967; doi:10.1371/journal.pone.0261482)
Supplement: S3 Appendix — A more detailed discussion of some technical aspects of our model. (PDF) [file pone.0261482.s005.pdf]

Appendix S3: Additional modelling details

The geometry of the model entails some considerations which are beyond the technical scope of the paper; however, we wish to discuss them for the more mathematically inclined reader. Firstly, since the modelled liquid crystals are droplets, topological constraints arise. Namely, the hairy ball theorem states that such liquid crystals have an Euler characteristic of $+2$, which means the total topological defect charge will be $+2$ [1]. In theory, this means that the orientational alignment would break down at the tips of the tactoid. In reality, in tactoids made up of Pf4 bacteriophages the phages curve around the tips of the tactoid as shown in the images by Tarafder et al. (2020) [2]. This means that the alignment is smooth and well-defined even at the tips. The hairy ball theorem still applies, which means that the tactoid will contain topological defects, but their influence on the diffusion of the antibiotics will likely be negligible. For this reason, and since we neglect the curvature of the tactoid, we do not consider the presence of the defects.
Secondly, liquid crystals are anisotropic structures. This means that certain of the material parameters will change in value depending on the direction along which it is considered. These parameters would therefore be tensors; however, our model uses the alignment and high aspect ratio of the phages to reduce the model to two dimensions. This way, only the isotropic directions of the liquid crystal are considered, and the material parameters are scalars.

References

[1] Nicola Carenza, Livio and Gonnella, Giuseppe and Marenduzzo, Davide and Negro, Giuseppe. Rotation and propulsion in 3D active chiral droplets. PNAS. 2019;117(44):22065–22070. doi:10.1073/pnas.1910909116.

[2] Tarafder AK, von Kügelgen A, Mellul AJ, Schulze U, Aarts DGAL, Bharat TAM. Phage liquid crystalline droplets form occlusive sheaths that encapsulate and protect infectious rod-shaped bacteria. PNAS. 2020;117(9):4724–4731. doi:10.1073/pnas.1917726117.
